# Successful conservative treatment of spontaneous intrathoracic esophageal perforation using a temporary covered esophageal stent with a check valve: a case report

**DOI:** 10.1186/s40792-019-0717-3

**Published:** 2019-10-24

**Authors:** Ryu Matsumoto, Ken Sasaki, Itaru Omoto, Masahiro Noda, Yasuto Uchikado, Takaaki Arigami, Yoshiaki Kita, Shinichiro Mori, Kosei Maemura, Shoji Natsugoe

**Affiliations:** Department of Digestive Surgery, Breast and Thyroid Surgery, Graduate School of Medical and Dental Sciences, Kagoshima University, 8-35-1 Sakuragaoka, Kagoshima-shi, Kagoshima, 890-8520 Japan

**Keywords:** Spontaneous intrathoracic esophageal perforation, Conservative treatment, Covered esophageal stent with a check valve

## Abstract

**Background:**

Spontaneous esophageal perforation is a potentially life-threatening condition with high morbidity and mortality rates. While surgical treatment has been employed for esophageal perforation, we have adopted conservative treatment with an esophageal stent for patients in a poor physical condition because we consider controlling sepsis and improving the physical status are the highest priorities; additionally, the surgical trauma could be fatal for these patients.

**Case presentation:**

A 60-year-old male complaining of left chest and back pain after vomiting was transferred to a local hospital. Computed tomography and chest X-ray examinations showed left tension pneumothorax, pneumomediastinum, and bilateral pleural effusion suspicious of spontaneous intrathoracic esophageal perforation. He was transferred to our hospital for further treatment. After arrival, he developed septic shock with acute respiratory failure. We considered that surgical treatment was too invasive and chose conservative treatment with an esophageal stent. Under general anesthesia, we first inserted a 20-Fr. trocar in the left posterior pleural space, and a large volume of the dark pleural effusion was discharged. We then performed endoscopy and found a pinhole perforation in the left posterolateral wall of the lower esophagus. We inserted both a silicon-covered esophageal stent with a check valve and a double elemental diet (W-ED) tube. We then inserted an 18-Fr. trocar into the left anterior wall. These procedures were performed less than 24 h after onset. As intensive medical care, the patient was administered broad-spectrum antibiotics and catecholamine. The two trocars and the W-ED tube were under continuous suction at − 5 cmH_2_O and at − 20 cmH_2_O every 30 s. On the 6th day, we inserted an additional thoracic drainage tube into the left pleura under CT guidance. The patient was discharged from the ICU to the general ward on the 7th day. We removed the stent almost triweekly, and the esophageal perforation was completely healed on the 45th day. He was discharged home on the 70th day.

**Conclusion:**

Conservative treatment with a temporary self-expanding covered stent with a check valve, sufficient drainage, and W-ED tube nutrition was useful and effective in this unstable case of spontaneous intrathoracic esophageal perforation.

## Background

Spontaneous esophageal perforation is a potentially life-threatening condition with high morbidity and mortality rates (ranging from 10 to 50%) [1, 2] that can be complicated with mediastinitis, thoracic empyema, and severe sepsis. Because it can be fatal if not treated appropriately at an early stage [3, 4], prompt diagnosis and appropriate treatment plans are mandatory. While surgical treatment, such as primary repair or debridement, has been employed for esophageal perforation [5], conservative treatment is limited to patients with a disruption in the mediastinum or between the mediastinum and visceral lung pleura, drainage of the cavity back into the esophagus, minimal symptoms, and minimal signs of clinical sepsis [6]. We have adopted conservative treatment with a temporary self-expanding silicon-covered stent rather than surgical treatment for patients in a poor physical condition because we consider controlling sepsis and improving the physical status are the highest priorities; additionally, the surgical trauma could be fatal for these patients [7]. Here, we present a case of spontaneous intrathoracic esophageal perforation with septic shock successfully treated conservatively with a temporary self-expanding silicon-covered stent with a check valve, sufficient drainage, and W-ED tube nutrition.

## Case presentation

**Fig. 1:**
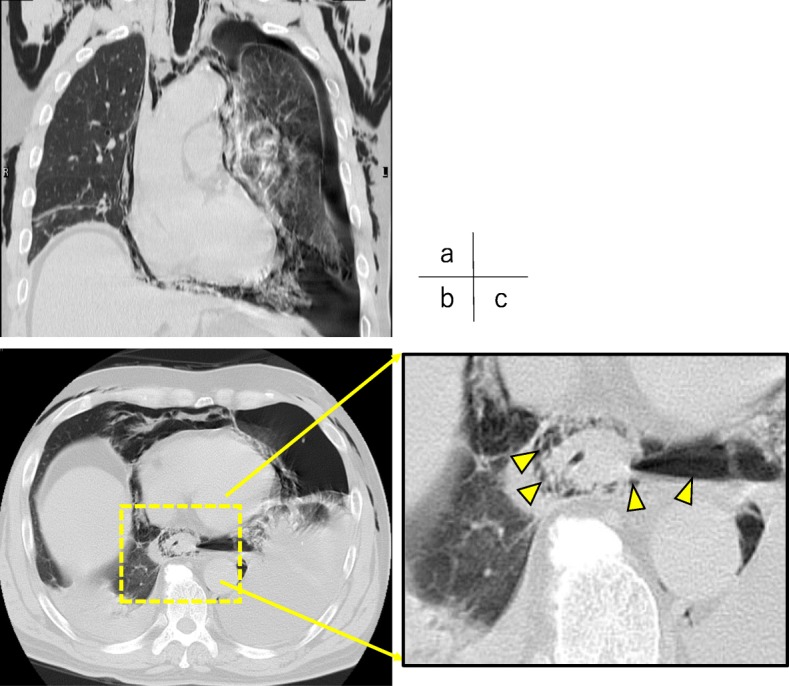
Preoperative chest CT findings. Chest CT revealed left tension pneumothorax (**a**) and pneumomediastinum around the lower esophagus (arrowheads) (**a**–**c**). **a** Transverse section. **b** Lower esophagus. **c** Enlarged view of the lower esophagus

**Fig. 2:**
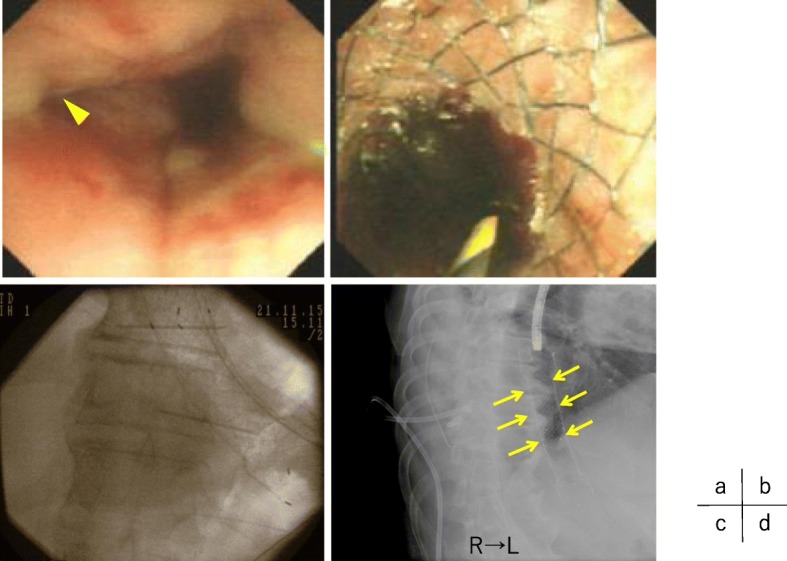
Esophageal stent placement. **a** Endoscopy revealed a pinhole perforation in the left posterolateral wall of the lower esophagus (arrowhead). **b**–**d** Esophageal stent placement (arrows) (**b** endoscopy, **c** fluoroscopy, and **d** chest X-ray)

**Fig. 3:**
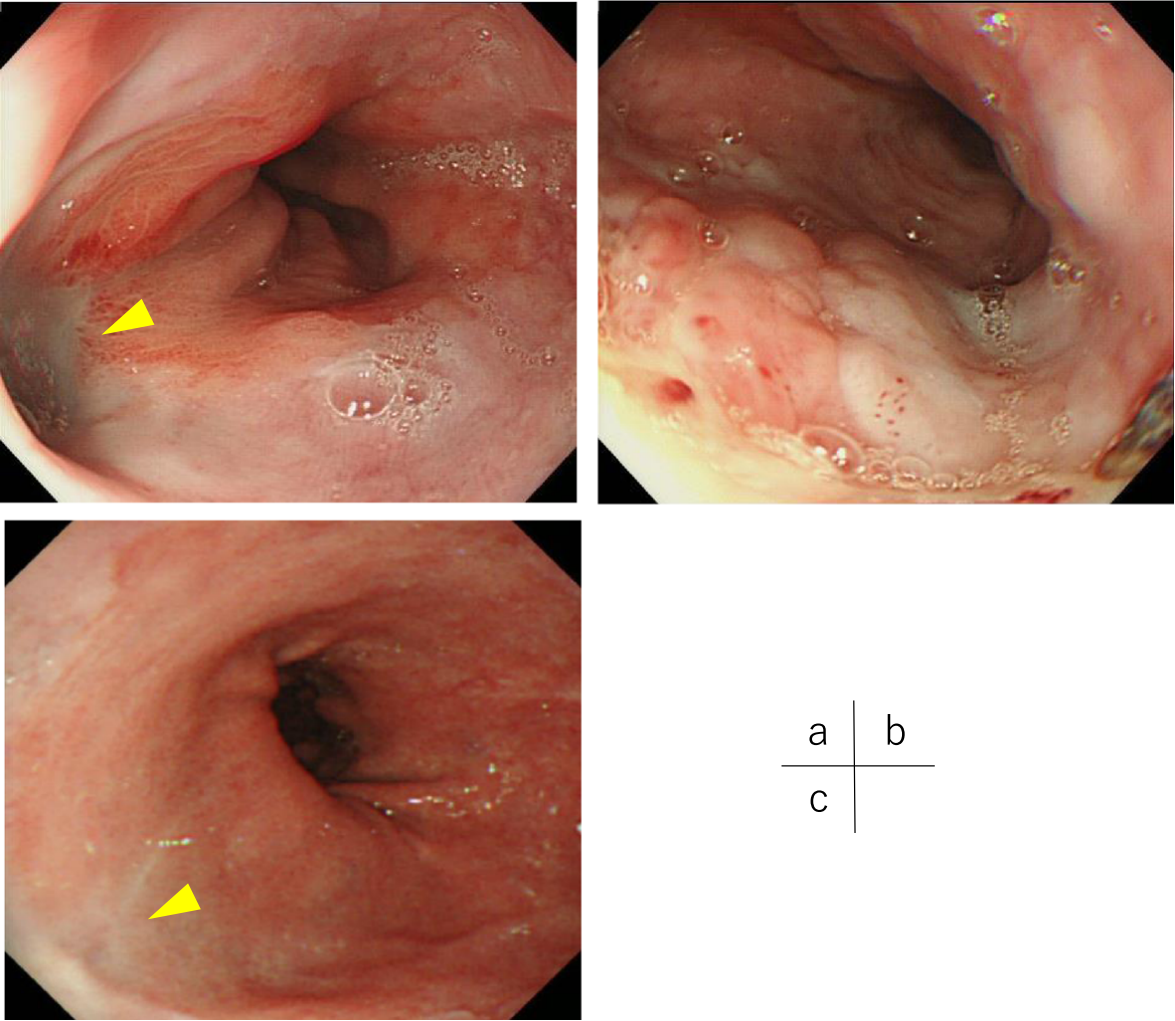
Complete healing of the esophageal perforation. **a** Healing of the esophageal perforation on the 45th day (arrowhead). **b** There was granulomatous tissue growth around the esophageal stent. **c** Complete healing of the esophageal perforation at 4 months (arrowhead)

A 60-year-old male complaining of sudden left chest and back pain after the severe vomiting of alcohol was transferred to a local hospital. Computed tomography (CT) and chest X-ray examinations showed the findings of left tension pneumothorax, pneumomediastinum, and bilateral pleural effusion, suspicious of spontaneous intrathoracic esophageal perforation (Fig. 1). A 20-Fr. trocar was inserted into the left chest pleural cavity, and cloudy dark pleural effusion with gastric juice was discharged. He was transferred to our hospital by a helicopter for further treatment. On arrival, the patient was tachycardic (113 bpm) and tachypneic (32 bpm) and showed signs of respiratory failure (SpO_2_ of 96% with oxygen at 8 L). He breathed with difficulty, and the breath sounds of the left chest were decreased. There was subcutaneous emphysema in the neck and chest. His face was pale, and his skin was cool and damp. The drain inserted in the previous hospital was removed accidentally during the transfer. His blood test results were as follows: total white blood cell count, 8600/L; platelet count, 229,000/L; C-reactive protein level, 25.68 mg/dl; blood urea nitrogen level, 27.9 mg/dl; and serum creatinine level, 1.36 mg/dl. Through the examinations, his general condition became worse and he began to show dramatic signs of septic shock. We diagnosed him with intrathoracic esophageal perforation and chose conservative treatment with esophageal stent and drainage of the chest and mediastinum rather than any aggressive surgical treatment because surgical trauma in such a critically ill patient could be fatal. We first transferred the patient to the operating room. The anesthetist administered an induction agent while applying cricoid pressure, and rapidly performed the intubation without positive pressure ventilation not to damage the perforation site of the esophagus (rapid sequence induction). Under general anesthesia, we inserted a 20-Fr. trocar into the left posterior pleural space under fluoroscopic guidance; soon, approximately 1300 cc of dark fluid with gastric juice was discharged, and then his vital signs became stable. We evaluated the location of the esophageal perforation by endoscopy and identified a pinhole in the left posterolateral wall of the lower esophagus (38 cm from an incisor) (Fig. 2a). We did not perform esophagography. We placed a silicon-covered stent with a check valve (HANROSENT®, M.I. Tech, Gyeonggi-do, Korea) at the esophagogastric junction (EGJ) to cover the area of the fistula (Fig. 2b–d). Next, we inserted a double elementary diet tube (W-ED® tube, Covidien, Japan) into the duodenum and an 18 Fr. trocar into the left anterior wall. The operative time was 58 min, and the bleeding volume was little. These procedures were performed less than 24 h after onset. As intensive medical care, the patient was administered broad-spectrum antibiotics and catecholamine. The bacterial culture of the blood did not represent any bacteria, but we firstly administered a broad-spectrum antibiotic and an antifungal drug, because our patient was susceptive of bacterial septic shock. Enteral nutrition with the W-ED tube was started on the next day. The two trocars and the W-ED tube were under continuous suction at − 5 cmH_2_O mmHg and at − 20 cmH_2_O every 30 s. On the 6th day, we inserted an additional thoracic drainage tube into the left pleura under CT guidance due to fluid accumulation close to the perforation site, and the patient was discharged to the general ward on the 7th day. We performed endoscopy to evaluate leakage using urografin and changed the esophageal stent almost triweekly. The fistula was healed on the 45th day (Fig. 3a) and the granulomatous tissue growth around the esophageal stent was found (Fig. 3b), and then the esophageal stent was finally removed on that day. On the 59th day, we removed the W-ED tube, and the patient began eating orally. The thoracic drainage tubes and trocars were removed, and he was discharged home on the 70th day. Endoscopy showed the complete healing of the fistula at 4 months (Fig. 3c).

## Discussion

Spontaneous esophageal perforation, especially in patients with septic shock, is a lethal condition, and a prompt diagnosis and appropriate treatment are mandatory for a successful clinical course [8, 9]. The esophagus lacks a protective serosal surface anatomically, and perforation of the esophagus can easily affect the surrounding tissue, leading to severe mediastinitis and emphysema of the pleural cavity [10]. The principles of managing esophageal perforation include controlling ongoing spillage from the esophagus, draining the pleural and/or mediastinal cavities, administering intravenous broad-spectrum antibiotics, performing gastric decompression, and providing enteral or parenteral nutritional support [9].

The treatment strategy for esophageal perforation is still controversial, especially regarding the control of spillage from the perforation site in patients with shock; in other words, whether surgical or nonsurgical management (conservative treatment) should be applied remains a matter of debate. Esophagectomy or primary repair is believed to be capable of eliminating the perforated esophagus as a source of sepsis and removing any underlying esophageal pathology [11, 12]. However, many patients develop severe postoperative sepsis and cannot be cured [10, 13]. Recently, some authors reported laparoscopy or camera-assisted surgery as a less invasive procedure than thoracotomy for treating spontaneous esophageal perforation [14–16], but these techniques require a highly skilled operator and a longer operative time, and closure of the perforation site after 24 h is more likely to lead to dysraphia [17].

On the other hand, conservative treatment, which avoids surgical trauma and allows drainage and control of sepsis, has been used for esophageal perforation [18], especially in critically ill patients [19, 20]. Vogel et al. reported an overall survival rate of 96% in 47 patients with esophageal perforation and emphasized that the aggressive treatment of sepsis and pleural fluid collection can prevent the need for major surgery [19]. In addition, the efficiency of self-expanding esophageal stents, such as the HANAROSTENT and SX-ELLA esophageal stents (ELLA-CS, Hradec Kralove, Czech Republic), in blocking the site of leakage has been reported, suggesting that they might help control sepsis and reduce mortality and morbidity [9, 21, 22]. Dasari et al. reported a technical success rate of stent placement of 91.4% and a healing rate of 81% [9]. To our knowledge, there have been only a few reports about the usefulness of “esophageal stent with a check valve” and “W-ED tube” for esophageal perforation. Oshiro et al. reported the successful management of staple line leakage at the esophagogastric junction after sleeve gastrectomy using the HANAROSTENT and its check valve at the distal end was effective to prevent gastroesophageal reflux [21]. Kimura et al. showed the usefulness of conservative treatment with fibrin glue injection and W-ED tube for esophageal perforation by a fish bone [23]. We believe using “esophageal stent with a check valve” and “W-ED tube” is beneficial to prevent the reflux of gastric juice to the fistula, especially to the fistula of the lower esophagus. Based on these reports, we think that the temporary placement of a self-expanding silicon-covered stent with a check valve is feasible for the treatment of critically ill patients with esophageal perforation. The use of esophageal stents in esophageal perforation has been reported to have some problems, such as the stent removal interval and stent migration [9]. An interval of esophageal stent replacement of approximately 3–8 weeks has been reported to avoid migration, fistula, and stenosis caused by granulomatous tissue growth [24], as well as the disruption of healed mucosa [9, 25]. The rate of stent migration has been reported to be 20.8% [9] and is expected to be higher with the use of stents in non-stricture-related etiologies, such as iatrogenic or spontaneous esophageal perforation. There have been few reports on methods for preventing stent migration. We experienced a patient who required esophagectomy at 3 months after insertion because of granulomatous tissue growth around the esophageal stent. Based on this valuable experience, we usually perform endoscopy to evaluate the presence of granulomatous tissue around the stent, esophageal ulcers, and stent migration every 3–4 weeks. We consider that if the perforation occurred in the lower esophagus, placing the stent at the EGJ is the most important for preventing stent migration. We first measured the length of the perforation site from the EGJ and then placed a sufficiently long stent at the EGJ. In the present case, we removed the stent every 3 weeks, and we did not experience any trouble with the esophageal stents, which were effective for healing.

In this case, we diagnosed spontaneous intrathoracic esophageal perforation based on the findings of bilateral pleural effusion, tension pneumothorax, severe broad pneumomediastinum, and contaminated drainage from the left thoracic cavity. We first transferred the patient to the operating room and then performed thoracic drainage under general anesthesia. As the general condition of patients with spontaneous intrathoracic esophageal perforation is often poor [26], it is necessary to prepare for unexpected vital changes. We did not perform esophagography because of the stress on the perforation site exerted by the ionic water-soluble media and the limited sensitivity (80–90%) [27]. Endoscopic visualization helps to identify the exact location of the perforation, extent of dehiscence, and viability of the esophageal mucosa [9]. Trained doctors perform endoscopy very carefully in a short time to avoid damaging the perforation site under CO_2_ insufflation to avoid worsening the pneumomediastinum. We used the HANAROSTENT stent to control the leakage from the fistula and the regurgitation of gastric juice to avoid outflow of the gastric juice through the fistula because this stent is removable, has a check valve, and is suitable for temporary applications. Furthermore, we placed the W-ED tube (16 Fr, 150 cm long, double lumen) into the jejunum for simultaneous jejunal nutrition and decompression of the stomach, which was also very effective for achieving early complete healing. When we suspected abscess formation based on the blood test and imaging findings, we immediately inserted an additional drainage tube under CT guidance.

In our institute, the first-line treatment plan for intrathoracic esophageal perforation, especially in patients with septic shock, has been conservative treatment to avoid surgical procedures, such as esophageal repair or esophagectomy, and often the placement of a temporary esophageal stent for spillage control. We treated 13 esophageal perforation cases from 2004 to 2019; among these, there were 4 cases, including this case, involving septic shock. We performed conservative treatment with esophageal stents, and we rescued all patients while avoiding surgical procedures [7]. Conservative treatment using esophageal stents with a check valve is effective in critically ill patients, but the further supporting evidence from additional cases is necessary for verification.

## Conclusions

We successfully saved this patient with spontaneous intrathoracic esophageal perforation and septic shock by conservative treatment with a temporary self-expanding covered stent with a check valve, sufficient drainage, and W-ED tube nutrition.

## Data Availability

Ethical approval was obtained from the Ethical Committee of Kagoshima University Hospital.

## Abbreviations

CT: Computed tomography
EGJ: Esophagogastric junction
W-ED: Double elementary diet tube
